# Effect of photobiomodulation therapy on painful temporomandibular disorders

**DOI:** 10.1038/s41598-021-87265-0

**Published:** 2021-04-27

**Authors:** Adila Aisaiti, Yanli Zhou, Yue Wen, Weina Zhou, Chen Wang, Jing Zhao, Linfeng Yu, Jinglu Zhang, Kelun Wang, Peter Svensson

**Affiliations:** 1 Department of Orofacial Pain & TMD, Affiliated Hospital of Stomatology, Nanjing Medical University, 1 Shanghai Road,Gulou District, Nanjing, 210029 Jiangsu People’s Republic of China; 2 Jiangsu Key Laboratory of Oral Diseases, Orofacial Pain & TMD Research Unit, Affiliated Hospital of Stomatology, Nanjing Medical University, Nanjing, People’s Republic of China; 3 Center for Sensory-Motor Interaction (SMI), Department of Health Science & Technology, Aalborg University, Ålborg, Denmark; 4 Section of Orofacial Pain and Jaw Function, Department of Dentistry and Oral Health, Aarhus University, Aarhus, Denmark; 5 Faculty of Odontology, Malmø University, Malmö, Sweden; 6 Scandinavian Center for Orofacial Neurosciences (SCON), Aarhus, Denmark

**Keywords:** Medical research, Clinical trial design, Randomized controlled trials

## Abstract

To evaluate the effect of photobiomodulation therapy (PBMT) on painful temporomandibular disorders (TMD) patients in a randomized, double-blinded, placebo-controlled manner. Participants were divided into a masseter myalgia group (n = 88) and a temporomandibular joint (TMJ) arthralgia group (n = 87) according to the Diagnostic Criteria for Temporomandibular Disorders (DC/TMD). Both groups randomly received PBMT or placebo treatment once a day for 7 consecutive days, one session. The PBMT was applied with a gallium-aluminum-arsenide (GaAlAs) laser (wavelength = 810 nm) at pre-determined points in the masseter muscle (6 J/cm^2^, 3 regions, 60 s) or TMJ region (6 J/cm^2^, 5 points, 30 s) according to their most painful site. Pain intensity was rated on a 0–10 numerical rating scale (NRS) and pressure pain thresholds (PPT) and mechanical sensitivity mapping were recorded before and after the treatment on day 1 and day 7. Jaw function was assessed by pain free jaw opening, maximum unassisted jaw opening, maximum assisted jaw opening, maximum protrusion and right and left excursion. Data were analyzed with a mixed model analysis of variance (ANOVA). Pain intensity in arthralgia patients decreased over time (P < 0.001) for both types of interventions, however, PBMT caused greater reduction in pain scores than placebo (P = 0.014). For myalgia patients, pain intensity decreased over time (P < 0.001) but without difference between interventions (P = 0.074). PPTs increased in both myalgia (P < 0.001) and TMJ arthralgia patients over time (P < 0.001) but without difference between interventions (P ≥ 0.614). Overall, PBMT was associated with marginally better improvements in range of motion compared to placebo in both myalgia and arthralgia patients. Pain intensity, sensory function and jaw movements improve after both PBMT and placebo treatments in myalgia and arthralgia patients indicating a substantial non-specific effect of PBMT.

## Introduction

Temporomandibular disorders (TMD) cover a number of clinical conditions affecting the temporomandibular joint (TMJ), masticatory muscles and related structures^1^. Pain is one of the most common and limiting clinical manifestations of such disorders^2^. The pathophysiology of pain in TMD patients is still not fully understood, however, peripheral and central sensitization of the trigeminal nociceptive pathways in addition to impairment in endogenous pain modulatory mechanisms influenced by genetic and epigenetic factors, and a wide and multifaceted range of environmental conditions such as for example bruxism, psychosocial distress, mood, sleep patterns etc. are encapsulated in the bio-psycho-social model for chronic pain^3,4^. Recent classification schemes have taken the step to include TMD pain as a primary type of chronic pain meaning that pain in itself has become the disease without a clear and identifiable cause whereas the secondary types of (TMD) pain are viewed as painful conditions which are caused by a disease or disorder and therefore represent a symptom rather than a disease^5,6^. In this scenario, causal treatment is not an option and palliative and non-invasive treatment modalities should be preferred. Several non-invasive treatment modalities have been used to relieve painful TMD conditions including occlusal splints, ultrasonography, pharmacologic-based therapy^7^, manipulative therapy^8^, oral motor exercise^9^, transcutaneous electrical stimulation, and photobiomodulation therapy (PBMT)^10^. Studies in specific TMD pain populations are, however, needed before strong recommendations can made.

PBMT has been widely used as an alternative or complementary treatment for TMD pain due to the non-invasive and safe features related to the low-intensity energy and wavelength characteristics^11,12^. The mechanism of photobiomodulation (PBM) is still debated but may be related to its potential influence on the synthesis, release, and metabolism of countless signaling substances involved in pain and analgesia^13,14^. Many prospective clinical trials have been performed to evaluate the efficacy of PBMT^11,13,15–17^, however, the results have been ambiguous due to the different laser parameters, lack of standardized operation procedures, lack of reliable methodology including placebo control, and small sample sizes. Therefore, more solid evidence is needed to evaluate the therapeutic effect of PBMT in TMD pain patients.

A standardized quantitative sensory testing (QST) developed by the German Research Network on Neuropathic Pain (DFNS) has been shown to be reliable in the orofacial region and upper and lower limbs^18–20^. The QST technique has the advantage of more objective and accurate evaluation of patients’ somatosensory function. In addition, a mechanical sensitivity mapping technique which has recently been used in the field of headache disorders such as migraine^21^ and chronic tension-type headache^22^ has been confirmed to have excellent reliability to demonstrate changes in somatosensory function and mechanical sensitivity in the masseter muscle and TMJ regions^23^.

Patients with TMJ pain often involve disorders of the articular surfaces, articular disk, bilaminar zone, joint capsule and ligaments, manifested as sound, limited mandibular movement and pain in these areas, while patients with muscle disorders often present with pain in the related muscle region, reduction of mandibular mobility and “pulling” sensations such as feeling of fatigue and bracing^24^. In addition to these differences in clinical manifestations, the mechanism of TMJ pain and myalgia may also be different^24^. Since there are different clinical manifestations and potentially different underlying pain mechanisms between TMD myalgia and TMJ arthralgia, studies may need to test the efficacy of interventions in both groups to obtain a better platform for evidenced-based recommendations for management. Therefore, the purpose of this study was to evaluate the effect of PBMT on myalgia and TMJ arthralgia patients by use of QST and pain sensitivity mapping techniques in a randomized, double-blinded, placebo-controlled manner.

## Results

**Figure 1:**
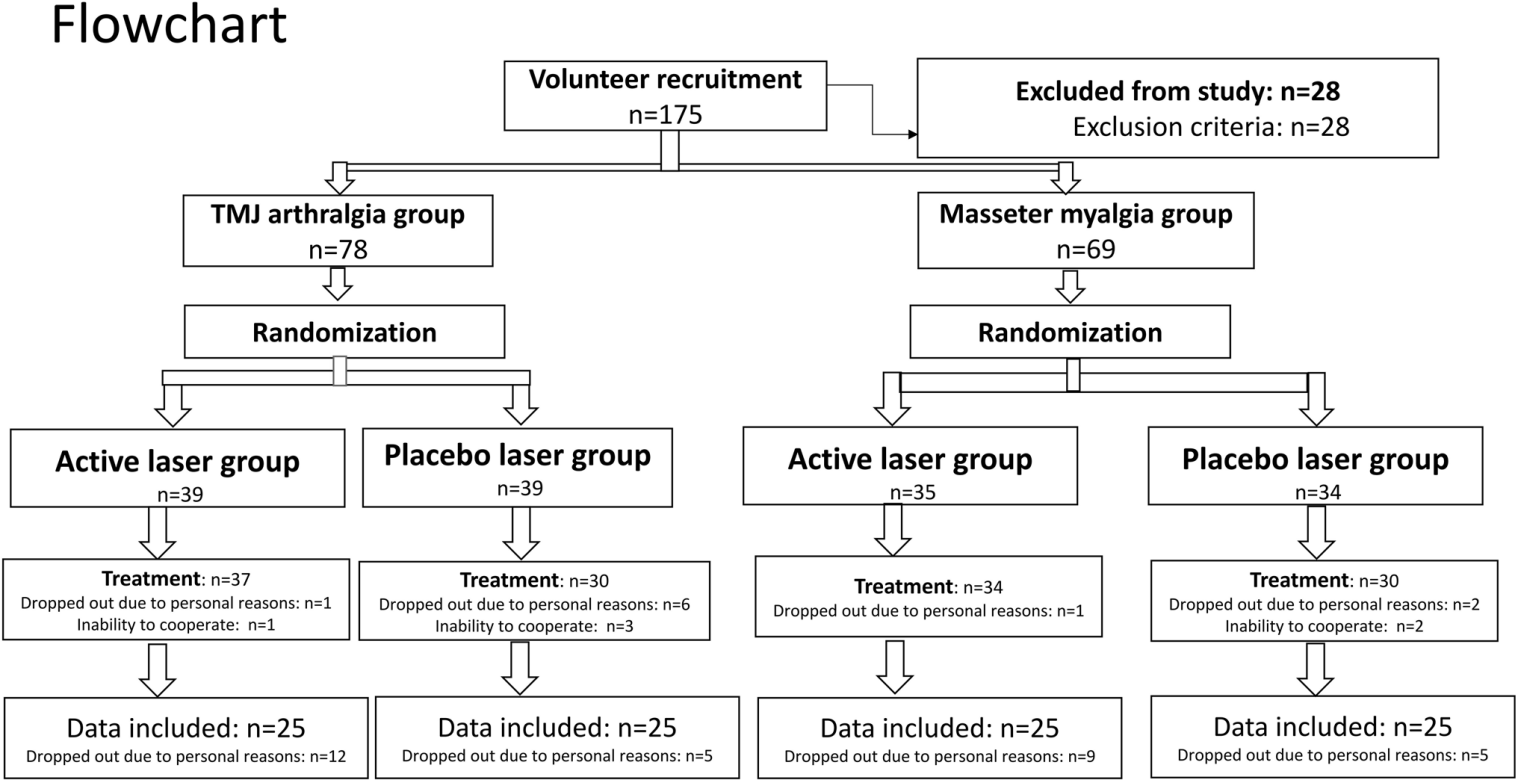
Flow chart of study. Personal reasons: include lack of time, work problem or reluctance to continue the experiment.

A total of 175 participants were initially enrolled into the study, 28 of them were excluded due to the exclusion criteria. 147 participants were randomized into 4 groups, while 16 participants dropped out due to personal reasons or inability to cooperate. In total, 100 participants completed all sessions in the study and 31 participants were lost in the follow-up during the study due to personal reasons (Fig. 1 (Supplementary Fig. S1)). Eight participants (massester myalgia: 3; TMJ arthralgia: 5) from PBMT group received occlucal splint therapy or pharmacology treatment for further treatment after experiment, while all participants in the placebo group received one week of real laser therapy for free, and 3 of them (masseter myalgia: 1; TMJ arthralgia: 2) received occlucal splint therapy at a later stage. No adverse effects were observed during and after the treatment period. The clinical characteristics of participants in each group are shown in Table 1. Regarding the treatment expectations (PBM/placebo: mean ± standard deviation = myalgia: 6.1 ± 2.4/5.4 ± 2.5; arthralgia: 6.9 ± 1.9/6.4 ± 1.7), no significant difference was found between PBM and placebo (myalgia: F = 1.058, P = 0.314; arthralgia: F = 0.933, P = 0.344).

**Table 1:** Clinic characteristics of participants in different groups.

Group	Intervention	Age		Gender		Pain intensity (NRS: 0–10)	Pain history			Pain characteristics	
		Mean	SD	Men	Women		≤ 1 month	1–6 month	≥ 6 month	Intermittent	Persistent
Masseter myalgia	PBM	34.8	11.4	6	19	5.3	10	6	9	13	12
	Placebo	33.2	13.6	8	17	4.4	6	13	6	16	9
TMJ arthralgia	PBM	35.0	12.2	4	21	5.2	8	7	10	15	10
	Placebo	34.8	13.2	6	19	4.1	7	10	8	13	12

PBM: photobiomodulation.

### Effect of PBMT on myalgia patients compared with placebo

**Figure 2:**
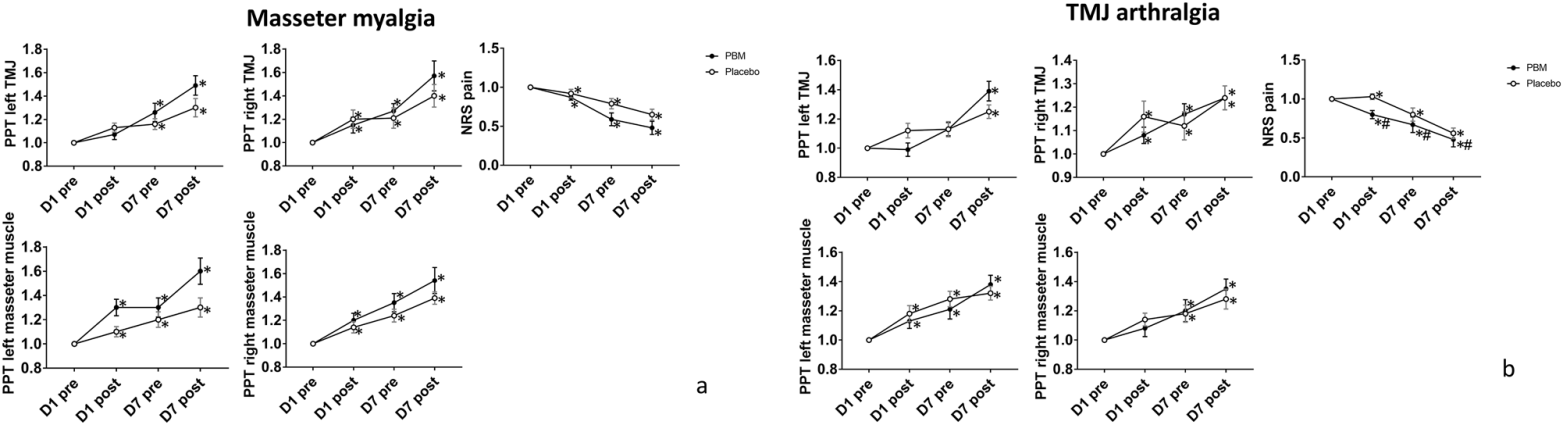
(**a**) The relative change of pressure pain thresholds (PPT) and NRS pain scores (mean and standard error) in masseter myalgia patients during the experiment (n = 50). Photobiomodulation (PBM) group (n = 25); Placebo group (n = 25). “*” indicates significant difference between D1-pre and other time points (P < 0.05). (**b**) The relative change of pressure pain thresholds (PPT) and NRS pain scores (mean and standard error) in TMJ arthralgia patients during the experiment (n = 50). Photobiomodulation (PBM) group (n = 25); Placebo group (n = 25). “*” indicates significant difference between D1-pre and other time points (P  < 0.05); “#” indicates significant difference between PBM and placebo (P < 0.05).

The relative changes of pressure pain thresholds (PPT) and numerical rating scale (NRS) scores of patients are shown in Fig. 2a (Supplementary Fig S2a). For the pain intensity, the main effect of time was significant for both PBMT and placebo treatment (F = 30.088; P < 0.001) without any difference between interventions (F = 3.492; P = 0.074), and no significant interactions between time and intervention (F = 2.551; P = 0.104); For pressure pain thresholds, PPTs at the masseter muscle and TMJ regions were significantly increased after both PBMT and placebo (Left TMJ: F = 28.893, P < 0.001; Right TMJ: F = 14.810, P < 0.001; Left masseter: F = 27.427, P < 0.001; Right masseter: F = 27.488, P < 0.001) without significant difference between intervention, (All ANOVAs: F < 2.544; P > 0.124) and no significant interactions between time and intervention was found (All ANOVAs: F < 2.542; P > 0.063).

**Figure 3:**
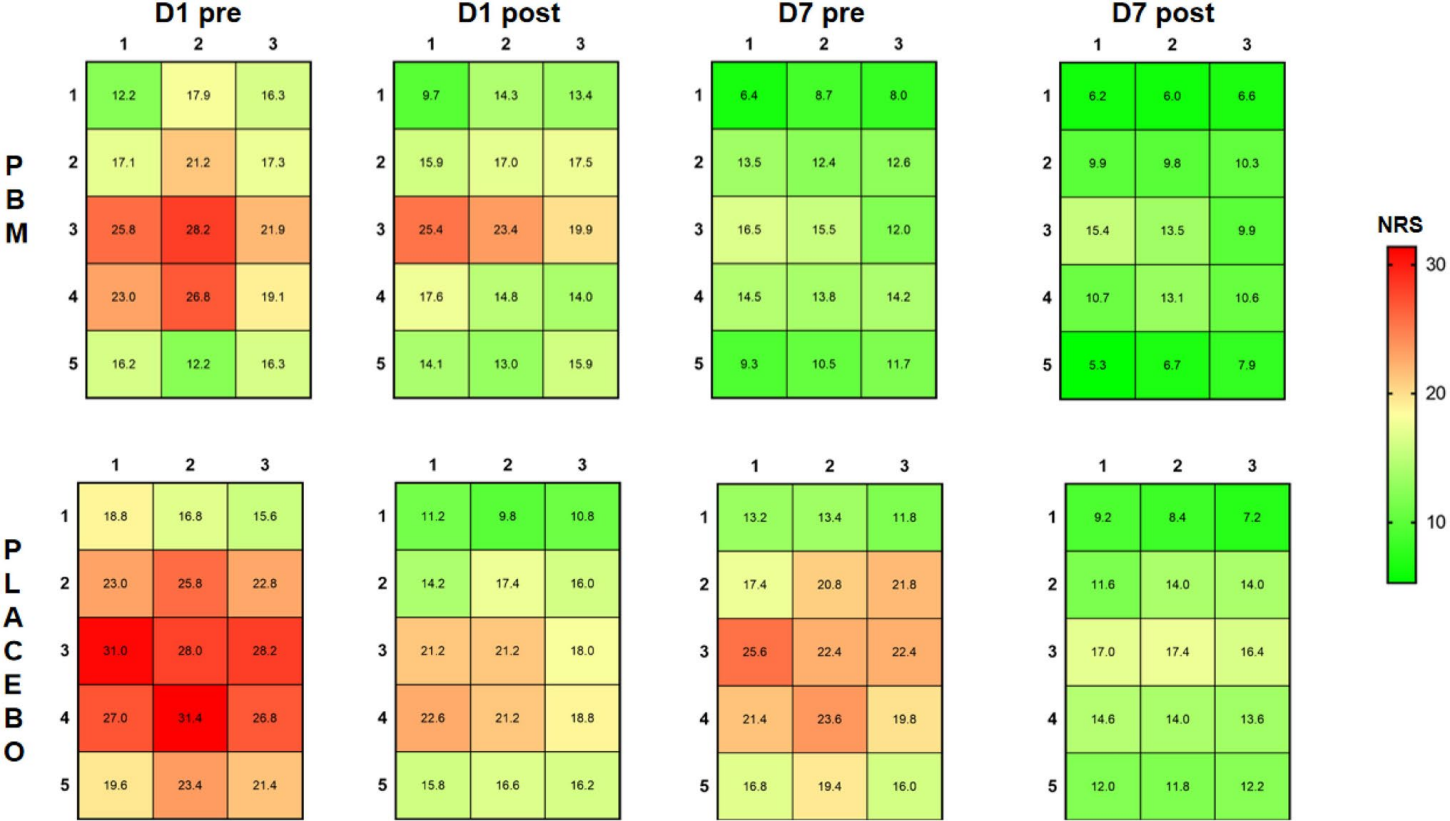
The mechanical sensitivity mapping of masseter myalgia patients before and after the treatment with photobiomodulation (PBM) (n = 25) and placebo (n = 25). NRS: numerical rating scale from 0–50–100 with 50 = moderate painful. Measurements were taken before and after treatment at Day 1 and Day 7 (D1 pre/D1 post; D7 pre/D7 post).

For mechanical sensitivity mapping, the changes of the mean NRS values of each grid point in every examination are presented in Fig. 3 (Supplementary Fig. S3). There were significant main effects of time on center of gravity (COG) coordinates and entropy for both PBMT and placebo treatment (COG-X: F = 15.639, P < 0.001, COG-Y: F = 16.029, P < 0.001, Entropy: F = 7.893, P < 0.001), but no significant main effects of intervention (COG-X: F = 0.718,P = 0.405; COG-Y: F = 0.631, P = 0.435; Entropy: F = 0.416, P = 0.525) nor interactions between time and intervention were found (COG-X: F = 1.815, P = 0.177; COG-Y: F = 1.115, P = 0.336; Entropy: F = 1.726, P = 0.185).

The results of analysis regarding jaw function assessment including pain free jaw opening, maximum unassisted jaw opening, maximum assisted jaw opening, maximum protrusion excursion and right and left excursion, COG coordinates and entropy of myalgia patients are shown in Table 2. For jaw movements, the participants in the PBMT group showed more improvement in mouth-opening and lateral excursion movement than those in the placebo group from Day 7-pre (pain free jaw opening: F = 4.919, P = 0.046, maximum unassisted jaw opening: F = 7.466, P = 0.022, maximum assisted jaw opening: F = 5.706, P = 0.021, left lateral excursion: F = 2.688, P = 0.016, right lateral excursion: F = 3.018, P = 0.041) and significant interaction effects between time and intervention were found in mouth-opening (pain free jaw opening: F = 5.191, P = 0.024, maximum unassisted jaw opening: F = 6.106, P = 0.012, maximum assisted jaw opening: F = 6.150, P = 0.008) and lateral excursion movements of patients( left lateral excursion: F = 7.826, P = 0.001, right lateral excursion: F = 3.839, P = 0.024).

**Table 2:** The descriptive statistics (mean ± standard deviation) and results of analysis regarding the test parameters of the masseter myalgia group during the experiment (n = 50).

Parameter	Intervention	D1pre		D1post		D7pre		D7post	
		Mean	SD	Mean	SD	Mean	SD	Mean	SD
**Jaw movement**									
Pain free jaw opening (mm)	PBM	28.0	6.9	29.5*	7.5	33.7*^#^	9.0	34.8*^#^	8.2
	Placebo	34.4	5.2	35.8	5.3	35.3	5.8	36.6	5.4
Maximum unassisted jaw opening (mm)	PBM	34.5	7.4	36.2*	7.4	37.6*^#^	7.9	38.8*^#^	7.8
	Placebo	40.2	4.7	41.2	5.2	40.3	5.4	41.1	4.8
Maximum assisted jaw opening (mm)	PBM	40.4	5.4	40.7	5.5	42.4^#^	5.5	43.0*^#^	5.3
	Placebo	42.3	4.7	42.5	4.5	41.9	4.1	42.5	3.8
Protrusion excursion (mm)	PBM	5.2	2.5	5.6	2.5	5.7	2.4	6.0*	2.5
	Placebo	4.6	2.1	5.0	1.8	4.3	1.8	4.8*	1.7
Left lateral excursion (mm)	PBM	8.2	2.2	8.0	2.3	9.0^#^	2.3	9.4*^#^	2.0
	Placebo	8.2	2.8	8.6	2.8	7.7	2.7	8.3	2.7
Right lateral excursion (mm)	PBM	6.8	3.2	7.0	2.7	8.1^#^	2.4	8.8*^#^	2.5
	Placebo	7.8	2.4	8.4	2.4	7.9	2.8	8.6	2.4
**Mechanical sensitivity mapping**									
COGX	PBM	1.2	0.9	1.1*	1.0	1.0	0.9	0.7*	0.7
	Placebo	1.4	1.2	1.1*	1.0	1.4	1.2	0.9*	0.8
COGY	PBM	1.9	1.5	1.6*	1.5	1.6	1.3	1.1*	1.0
	Placebo	2.2	1.8	1.7*	1.7	2.2	1.9	1.4*	1.3
Entropy	PBM	0.9	0.6	0.9	0.6	0.8	0.6	0.6*	0.6
	Placebo	1.0	0.5	0.8	0.5	0.9	0.6	0.7*	0.4

Photobiomodulation (PBM) group (n = 25); Placebo group (n = 25); “*” indicates significant difference between D1-pre and other time points (P < 0.05); “#” indicates significant differences between PBM and placebo (P < 0.05).

### Effect of PBMT on TMJ arthralgia patients compared with placebo

The relative changes of PPTs and NRS scores of patients are shown in Fig. 2b (Supplementary Fig S2b). For pain intensity, both groups showed a significant reduction over time (F = 25.469, P < 0.001). The NRS scores of patients were significantly lower in the PBMT group compared to the placebo group (F = 6.998, P = 0.014), but no significant interaction between time and intervention was found (F = 1.045, P = 0.350); For Pressure pain thresholds*,* the main effects of time were found in the PPTs of the right TMJ, left masseter muscle and right masseter muscle (right TMJ: F = 17.009, P < 0.001, left masseter: F = 29.878, P < 0.001, right masseter: F = 14.356, P < 0.001) without significant differences between intervention (All ANOVAs: F < 0.261; P > 0.614) or interaction between time and intervention (All ANOVAs: F < 1.129; P > 0.337). The PPTs in the left TMJ increased over time (F = 16.080, P < 0.001), but no significant treatment effect was found between the PBMT and placebo groups (F = 0.010, P = 0.923), however, the interaction between time and intervention was statistically significant (F = 3.753, P = 0.015).

**Figure 4:**
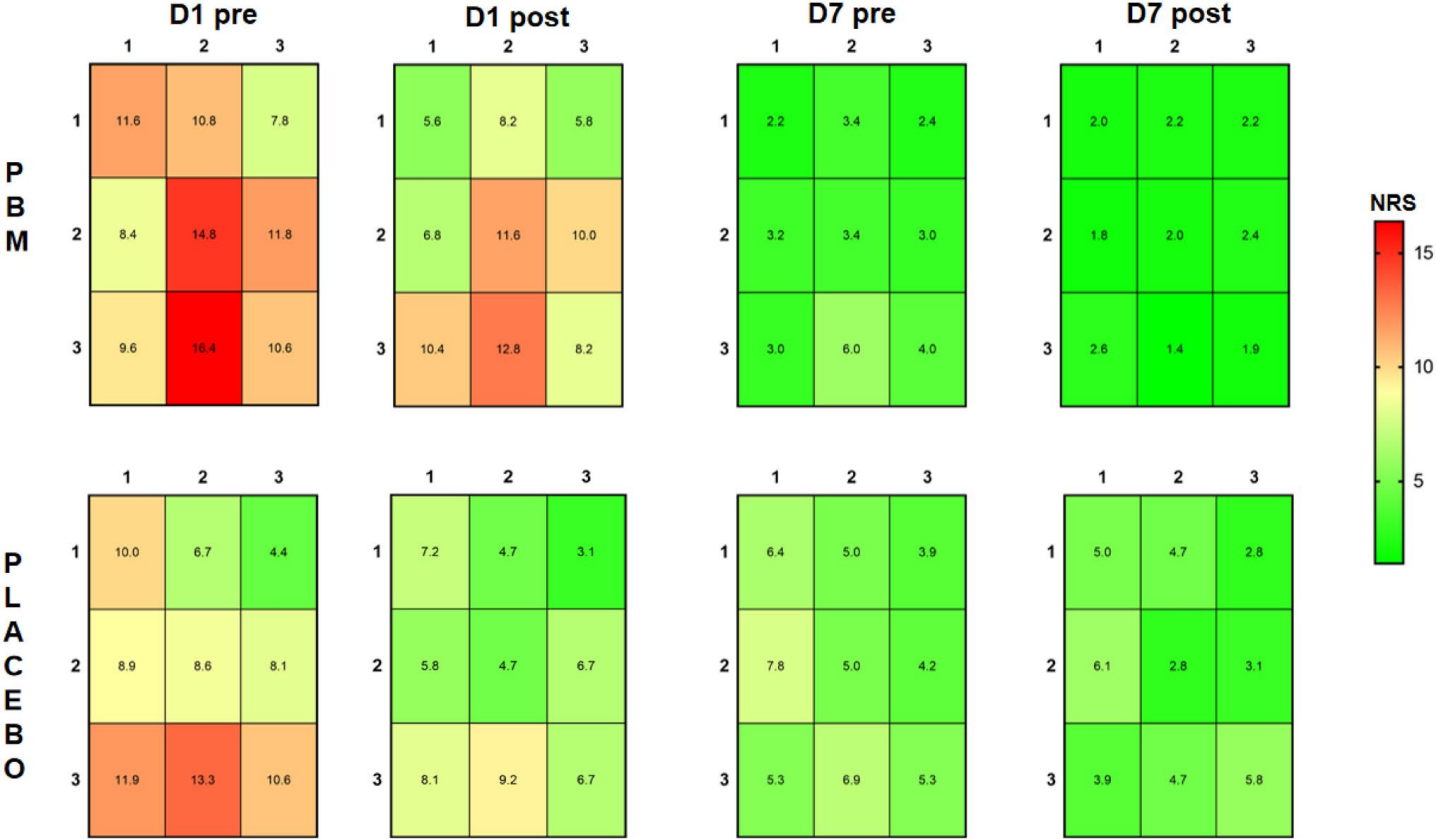
The mechanical sensitivity mapping of TMJ arthralgia patients before and after the treatment with photobiomodulation (PBM) (n = 25) and placebo (n = 25). NRS: numerical rating scale from 0–50–100 with 50 = moderate painful. Measurements were taken before and after treatment at Day 1 and Day 7 (D1 pre / D1 post; D7 pre/D7 post).

For mechanical sensitivity mapping, the changes of the mean NRS values of each grid in every examination are shown in Fig. 4 (Supplementary Fig. S4). Entropy values significantly decreased over time following both interventions (F = 91.749, P < 0.001) and were significantly lower after PBM compared to placebo at Day 7-post (F = 3.179, P = 0.041). A significant interaction effect between time and intervention of entropy was also found in the TMJ arthralgia patients (F = 3.347, P = 0.048). In terms of the COG coordinates, the COGX and COGY were significantly decreased over time (COGX: F = 15.369, P < 0.001, COGY: F = 21.877, P < 0.001) without a treatment difference (COGX: F = 0.268, P = 0.609; COGY: F = 0.116, P = 0.737) or interaction effect between time and intervention (COGX: F = 0.354, P = 0.651; COGY: F = 0.462, P = 0.573);

The results of analysis regarding jaw function assessment, COG coordinates and entropy of TMJ arthralgia patients are shown in Table 3. For jaw movements, the ranges of pain free jaw opening and right lateral excursion improved over time following both types of intervention (pain free jaw opening: F = 28.793, P < 0.001; right lateral excursion: F = 4.911, P = 0.013) while no significant main effect of intervention or interactions between time and intervention were found (All ANOVAs: F < 2.618, P > 0.101). Significant improvements in maximum unassisted jaw opening over time were found following both types of interventions (F = 10.034, P = 0.001), but more improvement was shown in the PBMT group compared to the placebo group from Day 1-post (F = 10.565, P = 0.041). No significant improvements were shown in maximum assisted jaw opening, maximum protrusion nor left lateral excursion movement following any of the interventions (All ANOVAs: F < 2.479, P > 0.113).

**Table 3:** The descriptive statistics (mean ± standard deviation) and results of analysis regarding the test parameters of the TMJ arthralgia group during the experiment (n = 50).

Parameter	Intervention	D1pre		D1post		D7pre		D7post	
		Mean	SD	Mean	SD	Mean	SD	Mean	SD
**Jaw movement**									
Pain free jaw opening (mm)	PBM	30.3	8.3	32.7*	7.2	36.8*	5.8	38.4*	6.3
	Placebo	33.0	6.9	35.4*	6.3	36.4*	6.5	37.8*	5.6
Maximum unassisted jaw opening (mm)	PBM	38.4	6.3	39.9^#^	5.9	41.1*^#^	5.2	42.1*^#^	5.4
	Placebo	40.8	4.9	40.8	5.4	40.8	5.2	41.2	4.8
Maximum assisted jaw opening (mm)	PBM	42.2	5.4	43.4	5.1	43.8	3.8	44.1	4.1
	Placebo	42.6	3.7	42.6	3.6	43.0	2.9	43.3	3.0
Protrusion excursion (mm)	PBM	5.0	2.2	5.1	1.8	5.2	2.0	5.6	1.9
	Placebo	5.5	2.0	5.7	1.8	5.6	0.8	5.8	0.9
Left lateral excursion (mm)	PBM	8.3	2.2	8.7	2.5	8.8	2.1	9.0	2.5
	Placebo	7.7	3.2	7.9	3.3	7.4	3.2	8.0	3.1
Right lateral excursion (mm)	PBM	7.8	2.9	8.5	2.2	8.8	2.1	9.2*	1.8
	Placebo	7.4	3.1	7.6	3.3	8.2	2.7	8.6*	2.7
**Mechanical sensitivity mapping**									
COGX	PBM	2.0	0.5	2.1	0.4	1.7*	1.0	1.5*	1.2
	Placebo	1.9	0.4	1.9	0.4	1.3*	0.9	1.3*	0.9
COGY	PBM	2.1	0.6	2.1	0.4	1.7*	1.0	1.4*	1.1
	Placebo	2.4	0.4	2.4	0.5	1.6*	1.1	1.6*	1.1
Entropy	PBM	0.5	0.5	0.4*	0.4	0.2*	0.3	0.1*^#^	0.3
	Placebo	0.5	0.5	0.4*	0.5	0.3*	0.4	0.3*	0.4

Photobiomodulation (PBM) (n = 25); Placebo (n = 25); “*” indicates significant difference between D1-pre and other time points (P < 0.05); “#” indicates significant differences between PBM and placebo (P < 0.05).

## Discussion

This randomized, placebo-controlled study indicated that both PBM and placebo interventions can reduce the self-reported pain intensity and improve the jaw function in TMD pain patients, but PBM showed marginally better effectiveness in patients with TMJ arthralgia and more advantages in improving jaw movement compared to placebo. Pain sensitivity of TMD patients appears to improve over time following both PBMT and placebo treatment without specific effects or differences between subgroups of TMD pain.

### Putative effects of PBMT on pain

As a non-invasive and non-pharmacological modality, PBMT has been suggested to be effective in improving mandibular movements and promoting analgesia in TMD pain patients^10,13,25–32^. The hypothesis on PBM effects mainly covers three aspects: modulation of inflammatory processes, alteration of excitation and nerve conduction in peripheral nerves, and release of endogenous endorphins. The effects of PBMT on inflammatory processes include changes in biochemical markers (histamine, prostaglandin E2, interleukin-1, tumor necrosis factor-β), altered distributions of inflammatory cells, reduced formation of edema, hemorrhage, and necrosis^33^. The thinly myelinated A-σ and unmyelinated, slow-conducting C fiber distributed under the epidermis form the peripheral nerve endings of nociceptors can be activated by noxious stimuli leading to propagation of action potentials. When the laser energy is absorbed by the skin, these action potentials are believed to be inhibited, causing changes in activation threshold of the nerve fibers and a decrease in the release of pro-inflammatory neuropeptides (i.e., substance P)^34^. The increase of serotonin and endorphin levels is a further putative mechanism of pain relief^35^. Although these putative molecular and biological mechanisms underlying of PBMT is far from complete, it is commonly believed that Cytochrome c Oxidase (CCO) in the mitochondrial respiratory chain serves as the primary photoceptor^36^. Since the action spectrum of PBMT matches the absorption spectrum of CCO, after absorption of photons by CCO, inhibitory Nitric Oxide (NO) which is non-covalently bound to the heme and Cu centers of CCO and competitively blocks oxygen at a ratio of 1:10 can be dissociated, and the activation of mitochondrial respiration can lead to increase of mitochondrial membrane potential (MMP), cyclic adenosine monophosphate (cAMP), oxygen resumption and ATP production^37^. NO is known as a vasodilator, which can stimulate the soluble guanylate cyclase to form cyclic-GMP (cGMP) and activates protein kinase G, which leads to reuptake of Ca^2+^ and opening of calcium-activated potassium channels, causing the relaxation of blood vessels and lymphatic vessels^38^. The mediated activation of transient receptor potential (TRP) ion-channels is also considered as one of the mechanisms of action of PBM. After laser irradiation, these cation channels will open and depolarize the cell membrane, the increase of intracellular Ca^2+^ can lead to the release of histamine to promote tissue healing^39,40^. Besides that, the lipid peroxidation of cell membranes can also lead to a generation of reactive oxygen species (ROS)^41^. ROS can activate the transcription factor nuclear factor kappa B (NF-kB), which regulates the expression of genes related cellular functions such as inflammatory-induced response and survival^42^. The activation of NF-kB can be induced by PBM to enhance gene transcription that leads to cell proliferation and migration, to reduce cell death and enhance neurological function^37^. Although the evidence for the mechanism of action of PBM is mounting, there continues to be controversies about the clinical effectiveness of PBMT in TMD pain patients due to lack of standardization of laser parameters and operating procedures, as well as lack of high-quality and sufficiently operationalized outcome measures. Therefore, the present study applied reliable and validated QST measures and comprehensive pain mapping techniques to provide more objective and robust evidence to evaluate the therapeutic effect of PBMT stimulation.

### Laser parameter

The effectiveness of PBM is related to the laser irradiation parameters including wavelength, power, power density, energy density, time and total energy^35^. In previous studies, it has been shown that lower doses of light are more effective than much higher doses due to a biphasic dose response curve exhibited by PBM^43,44^. In clinical practice, multiple PBM treatment protocols are available for TMD patients, after reviewing all available therapeutic regimens, a recent systematic review suggested an evidence-based protocol for clinical PBM administration for these patients^45^. According to this review, the application of GaAlAs diode laser, wavelength 800–900 nm, 100–500 mW and energy density lower than 10 J/cm^2^, twice a week for 30 days showed the best results for pain relief and improvement of mandibular movements^45^. To achieve the same total dose as the recommendation^45^ and minimize the possibility of spontaneous remission, we modified the treatment application to once a day for 7 consecutive days.

### Outcome measures

In many previous studies on PBMT in TMD pain patients the focus has been on jaw movements or self-reported pain intensity, only a few studies have determined changes in patients’ somatosensory function using more objective testing methods^46,47^. For example, simple rating scales like a NRS or VAS (Visual Analogue Scale) are the most popular way to evaluate the self-reported pain intensity in clinical practice; however, this approach may be an oversimplification of complex biopsychosocial pain problems and possibly result in an underestimation or overestimation of pain^46^.

Pressure algometry is part of the QST technology and is the most-commonly used method to detect pressure sensitivity and static mechanical allodynia in deep tissues^19^. Pressure algometry delivers both a stable, reliable and quantifiable pressure through a flat base applied to the dermal surface^48^. The force is generally applied as a gradual increased pressure stimulus by the examiner, and the stimulation is stopped once the test participant presses a button. The value of interest is typically defined as the pressure pain threshold (PPT) which is minimum pressure that the participant report to be consistently just barely painful^49,50^. PPT has been shown to be reliable for evaluation of deep pain sensitivity in e.g., the masseter muscle and TMJ regions^18–20^. In addition, a pain mapping technique has also been demonstrated to have excellent reliability in assessment of the spatial aspects of mechanical sensitivity in the masseter muscle area and TMJ regions^23^. This method is considered as a valuable approach for a more comprehensive investigation of the pathology of somatosensory system, and it can provide an overall description of how somatosensory function varies with time or change in sensitivity among multiple test sites^51^. Therefore, the pain mapping technique, which was performed with a simple quantitative palpometer in this study, is one of the main novelties of this experiment and demonstrated significant changes in deep pain sensitivity across the masseter and TMJ regions following both PBM and placebo interventions while PBM showed more advantage than placebo in TMJ arthralgia patients.

### Effects of PBMT on masseter myalgia

The present study showed that the self-reported pain intensity in patients with masseter myalgia was decreased and the somatosensory sensitivity was improved (less sensitive) following the intervention with both PBM and placebo, which are consistent with previous studies^52,53^. The present study found that the jaw movement of PBMT group showed more improvement than those in the placebo group, which is also corroborated by other studies^54–56^. The effectiveness of placebo in this study can also be related to the activation of endogenous opioids and neural mechanisms of pain modulation^57–59^. Pain modulation is, indeed, influenced by many factors, such as psychological issues, memory of pain experience, hope of health restoration, educational level and professional-patient relationship etc.^60–62^. The sham-laser treatment provided in the placebo sessions may have amplified the psychological impact and anticipation of treatment effects in myalgia patients which is reflected in similar expectations scores between PBM and placebo. Furthermore, a good relationship between the professionals and patients in the present experiment may also contribute to the improvement following the placebo intervention. From this perspective, we can consider that although the therapeutic effect of PBMT has been indicated in animal studies^63,64^, in human-beings the cognitive aspects of the placebo intervention is as important as the putative biological effects following PBMT in mediating an hypoalgesia effect in TMD pain patients with a sub-diagnosis of myalgia.

As for the results of pain mapping in myalgia patients, it was clear that patients showed the highest NRS scores around the middle part of the masseter muscle, while the lowest NRS value was found near bony structures and in the periphery of the masseter muscle consistent with previous studies^23^. Interestingly, the NRS pain scores decreased, COG coordinates shifted, and entropy values decreased as a reflection of a decrease and more homogenous muscle pain sensitivity following both types of interventions. This finding again questions the specificity of PBMT in patients with myalgia.

### Effects of PBMT on TMJ arthralgia

In the present study, PBM was found to have some minor advantages compared to placebo in terms of decreases in self-reported pain intensity and increases in maximum unassisted jaw opening as well as improvement in mechanical sensitivity in patients with TMJ arthralgia. However, in Emshoff et al.’s study^17^, the self-reported pain relief following PBM and placebo were both decreased but with no significant difference between groups. It should also be acknowledged that the present differences between PBM and placebo are small and perhaps without a robust clinical importance. Similar to the findings in myalgia patients, the pain mapping results indicated lower entropy scores following PBMT in TMJ arthralgia suggesting a normalization of the pain sensitivity in the TMJ region. It can be speculated that this effect is mediated by a combination of endogenous pain modulation mechanisms related to a placebo response and a peripheral component related to the putative therapeutic effect of PBMT on peripheral sensitization of the TMJ region. Direct measures of the biological markers in the TMJ would add more information to the understanding of PBMT effects in future studies.

### Limitations of the study

First, it is important to notice that the standardized operating procedure in the scientific literature about the dose and protocols of PBMT for TMD pain patients has not reached a consensus, however, we followed the best available evidence in terms of stimulation parameters. The present results cannot be extrapolated to all types of PBMT for different types of TMD pain patients. Second, it should also be mentioned that only the immediate effect of PBMT was evaluated in this study and the sample size was relatively small partly because of dropouts during the treatment procedure—a well-known challenge in RCT studies. We recommend that multicenter studies are performed in well-defined TMD patient groups (e.g., in accordance with the ICOP criteria) and with larger sample size and reliable and valid outcome measures including the proposed techniques from this study. Nevertheless, it should also be mentioned that we applied rigorous diagnostic criteria to subtype TMD pain patients and this is the first study to investigate PBMT in both TMJ arthralgia and myalgia patients.

## Conclusions

A striking finding in the present experiment was that both PBM and placebo interventions were associated with significant improvement over time and with only subtle differences between myalgia and arthralgia patients. PBMT seemed slightly more effective in terms of a decrease in self-reported pain intensity and improvement of the mechanical sensitivity in patients with TMJ arthralgia while PBM was more effective in terms of improvement of jaw movements in patients with masseter myalgia. Overall, these findings suggest that specific pain management should be tailored to the different subtype of TMD pain and that PBMT may involve substantial placebo effects.

## Materials and methods

### Study participants

A total of 175 TMD pain patients (68 men and 107 women, aged from 18 to 60 years old) were involved into the study from April 20th, 2020 to September 30th, 2020. The patients were recruited from the Department of TMD & Orofacial pain, Affiliated Hospital of Stomatology, Nanjing Medical University, P. R. China. The sample size was calculated a priori based on the detection of a minimum clinically relevant difference of 25% at an α level of 0.05 and 80% power (i.e., the risk of a type I and type II error was 5% and 20%, respectively). However, considering an anticipated 25% dropout rate, a total of 131 participants were involved and 100 patients were included for data analysis. All patients were examined and diagnosed with Diagnostic Criteria for Temporomandibular Disorders (DC/TMD)^65^ by trained examiners and medical histories were collected. Inclusion criteria were: TMD patients aged from 18 to 60 years old reporting unilateral pain in the masseter muscle or TMJ regions lasting at least 2 weeks without receiving alternative treatments in the past 3 months. The pain intensity assessed on a 0–10 NRS during manual palpation should be ≥ 4. Exclusion criteria were: history of orofacial trauma that affects normal somatosensory function; history of serious or chronic systemic diseases (e.g., trigeminal neuralgia, burning mouth syndrome or systemic musculoskeletal pain disorders such as fibromyalgia, or symptoms of rheumatoid arthritis); use of alcohol or caffeine within 24 h of the test day, and/or any mental disorders.

According to the exclusion criteria, 28 patients were excluded, 78 patients (50 women, 28 men; mean age ± SD: 34.9 ± 12.7 years) were recruited into the TMJ arthralgia group, and 69 patients (46 women, 23 men, mean age ± SD: 34.0 ± 12.6 years) were recruited into the masseter myalgia group (Fig. 1 (Supplementary Fig. S1)). Patients in both groups were randomly assigned to be treated with PBM or placebo according to the results of computer randomization program which was put in an opaque envelope. An individual unassociated with the study prepared the envelopes. Randomizations sequence was created using Stata version 9.0 statistical software and was stratified with a 1:1 allocation using block sizes of 4, 5, 6.

### Study protocol

A randomized, placebo-controlled, double-blinded clinical trial was conducted by two examiners. Examiner 1 was responsible for the examinations of participants before and after treatment and was blinded to which treatment the patient received, while the examiner 2 completed the randomization and treatment. Participants in both groups were randomly assigned to intervention with PBM or placebo once a day for 7 days. A specialist from the department of orofacial pain and TMD monitored the conditions of the participants during the whole study to take effective measures once the adverse effects occur.

**Figure 5:**
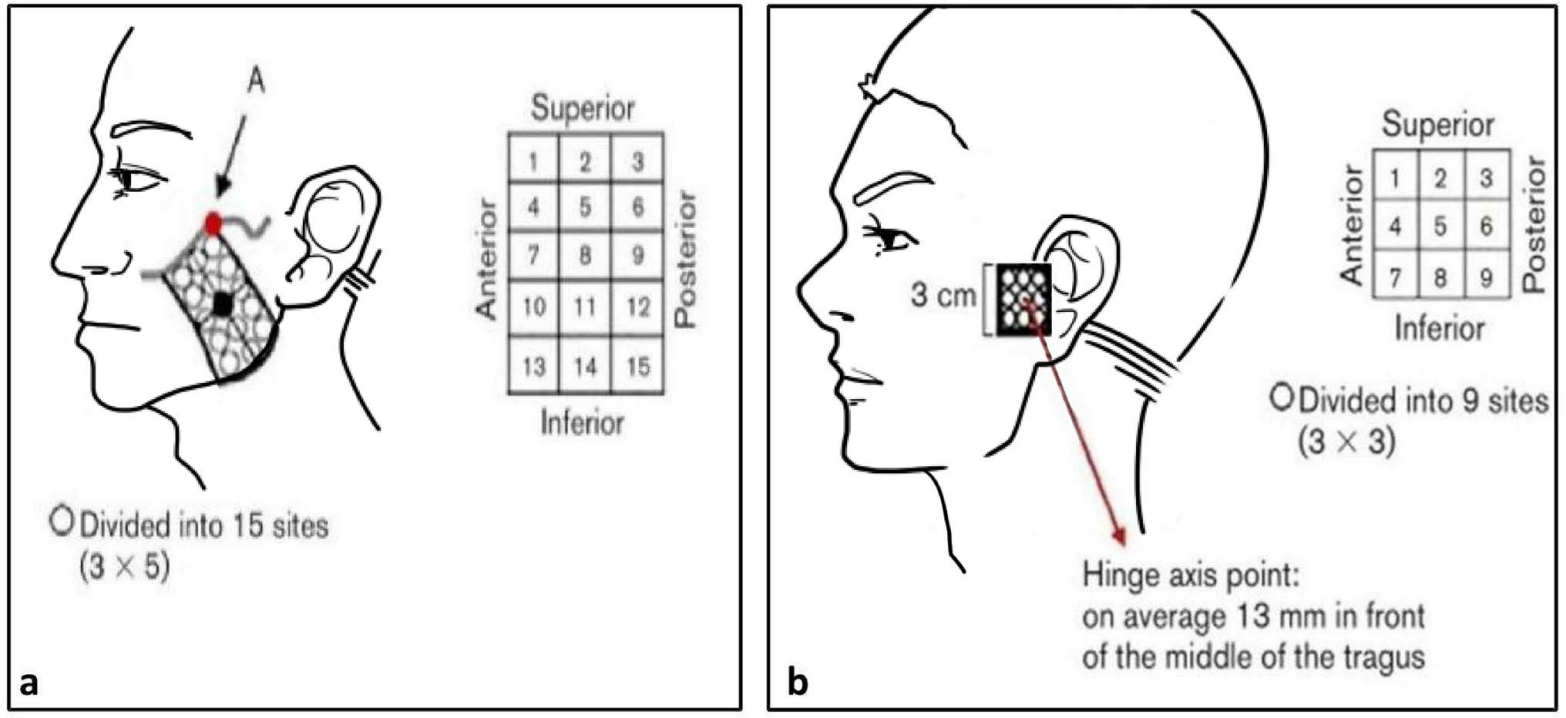
A quantitative mechanical palpometer with 1.0-kg pressure force was applied to 15 grids of masseter muscle (**a**) and a palpometer with 0.5-kg pressure force was applied to 9 grids of TMJ region (**b**). Point A = intersection point between the superior margin and posterior margin of the superficial masseter muscle. In the TMJ region the hinge axis point was on average 13 mm in front of the middle of the tragus.

All the examinations and treatment were performed in a reserved room with suitable temperature, and free from sound interference. During the treatment session, the participants were seated upright on a comfortable office chair to measure their jaw movement. Then, they were asked to rate the pain intensity with the use of a numerical rating scale 0–10 (NRS). After that, they were required to lie down in the dental chair, and examiner 1 measured the PPT values bilaterally at the center of the masseter muscle and at the hinge axis points of the TMJ regions (Fig. 5b (Supplementary Fig. S5b)). Pain sensitivity maps over the painful masseter muscle area and TMJ region were also evaluated by use of quantitative palpation^23^. There were 15 test points (Fig. 5a (Supplementary Fig. S5a)) above the superficial masseter muscle while the TMJ region included 9 test points (Fig. 5b (Supplementary Fig. S5b)). The NRS values reported by the patients at the test points after palpation were used as grids values to create a sensitivity map. Besides that, patients’ expectations to the allocated treatment were recorded with the use of a 0–10 NRS with 0 indicating “expectation of no treatment effect” and 10 indicating “expectations of the most imaginable effect”. All the above tests were assessed before (Day 1-pre) and after (Day 1-post) the treatment on day 1 and day 7 (Day 7-pre, Day 7-post).

### Laser and placebo treatment

According to the concept of “Optical window”^35^ and the evidence-based protocol for PBM administration^45^, a gallium-aluminum-arsenide (GaAlAs) laser (Pilot, CAO group, America) (wavelength = 810 nm) was used for PBMT and placebo treatment. The laser application was performed at pre-determined points on their masseter muscle area or TMJ region of their painful site: the masseter muscle (3 regions: superior, middle, inferior) and TMJ region (4 points forming a cross and one central point)^46^. Since the area of the masseter muscle was different in each individual and limited by the size of the laser light spot, every irradiation region of the masseter muscle was irradiated from anterior to posterior with approximately three to four irradiated circles to ensure all the test points were involved and received equal irradiation. The treatment application was once a day for 7 consecutive days. The PBM stimulation was applied in the continuous emission mode and did not have direct contact with the patient’s skin or mucous membrane, and irradiated the treatment site in a circular manner with a special trumpet-shaped handle. The irradiation parameters used were as follows: operating frequency = 10 Hz, laser optical power = 3 W, diameter of light spot = 2 cm, the distance between spot and optical fiber = 3 cm; for masseter muscle: energy density = 6 J/cm^2^, measurements of the output power in irradiated area = 100 mW, time per region = 20 s, total time = 60 s, for the TMJ area: energy density = 6 J/cm^2^, measurements of the output power in irradiated area = 100 mW, time per point = 6 s, total time = 30 s. The placebo treatment was done exactly in the same manner as the PBMT at the same points for the same time duration but without power output. During the treatment procedure, both participants and the professional responsible for the treatment were wearing special glasses for eye protection.

### Primary outcome measures

#### Pain intensity recording

Pain intensity was assessed by means of a 0–10 NRS where 0 indicates “no pain” and 10 means “the most pain imaginable”. Patients were instructed to rate the present pain intensity, and the NRS score was used to represent the intensity of patients’ pain at the time of evaluation.

#### Jaw movement measurement

Jaw movement including pain free jaw opening, maximum unassisted jaw opening, maximum assisted jaw opening, maximum protrusion excursion, left lateral excursion, and right lateral excursion were assessed during each examination per the DC/TMD instructions. The distance between the midline of upper and lower central incisors was measured with a ruler.

### Secondary outcome measures

#### Pressure pain thresholds (PPT)

PPTs were measured by a hand-held electronic pressure algometer (Algometer; Medoc, Ramat Yishai, Israel) with a probe diameter of 8 mm. The pressure was increased at a rate of 30 kPa/s^66^. The PPTs were measured bilaterally at the center of the masseter muscle and hinge axis points of the TMJs. Each site was tested for 3 times and the average was calculated and used for the statistical analysis.

#### Mechanical sensitivity mapping

A quantitative mechanical palpometer (Palpeter, Sunstar Suisses SA) was used to perform standardized palpation of the painful masseter muscle and TMJ regions to form a mechanical sensitivity map and analyze the pain intensity at different and multiple test points. The circular metal rod of the palpometer was 10 mm in diameter and made of aluminum. There was a hole at the other end of the palpometer to let the stamp-tapering end pass through. The palpometer was held perpendicularly to the surface skin of the participants with the examiner’s thumb and middle finger. The examiner would detect the tapered end with the index finger as the correct force was applied^23^.

In order to investigate the mechanical sensitivity maps, the superficial part of the masseter muscle was divided into 3 × 5 grids (Fig. 5a (Supplementary Fig. S5a)) while the TMJ region was divided into 3 × 3 grids (Fig. 5b (Supplementary Fig S5b))^23^. The palpometer with a 1.0-kg pressure force was applied to each of the 15 grid points at the masseter muscle, and a palpometer with 0.5-kg pressure force was applied to the 9 grid points of the TMJ region in a randomized order in accordance with the (DC/TMD) guidelines for manual palpation. All test sites were stimulated for approximately 2 s during each measurement. After each measurement, there was a 10-s interval for the participants to rate the perceived pain intensity of the stimulus on a 0–100 NRS which 0 meant “no pain”, 50 was defined “moderate painful”, and 100 “the most pain imaginable”^67^. The COG coordinates (x = anterior–posterior direction and y = superior-inferior direction) were defined as: $\sum Xi*grid \; value\left(i\right)/\sum grid \; value(i)$; $\sum yi*grid \; value\left(i\right)/\sum grid\; value(i)$; The 0–100 NRS values were used as grid values^68^. Shannon entropy was also calculated to represent the complexity and diversity of patients’ mechanical sensitivity^69,70^.

### Data analysis

In order to ensure the baseline was comparable and minimize the impact of patient-related factors such as individual cognitive differences, the way and time of enrollment, the different stages and duration of the disease, all parameters except entropy values, which was presented as the absolute difference (Tn-T0) were otherwise expressed as relative changes from baseline (Day 1-pre) (TN/T0) and logarithmic transformed to normalize the data distribution. Then, the calculated absolute difference and relative change from baseline were used for data analysis. A mixed model analysis of variance (ANOVA) was used to assess the main effects of time (repeated factor), intervention (between group factor) and interactions between factors. A significance level of 5% was applied.

All statistical work was performed using the Statistical Package for Social Sciences version 20 (SPSS, IBM). The figures and tables presented in this study were produced by Prism 7.0 software.

### Ethics approval and consent to participate

All procedures performed in studies were in accordance with the 1964 Helsinki declaration and its later amendments or comparable ethical standards. Confirms that informed consent was obtained from all participants and/or their legal guardians. The study was approved by the Nanjing Medical University Research Ethics Committee (PJ2019-005-01), and registered on the WHO international clinical trial registry platform (20/04/2020, ChiCTR2000032104).

### Consent to publish

Confirms informed consent was obtained to publish the information in an online open access publication.

## Supplementary Information

Supplementary Figure S1.

Supplementary Figure S2a.

Supplementary Figure S2b.

Supplementary Figure S3.

Supplementary Figure S4.

Supplementary Figure S5.

## Data Availability

The datasets generated during and/or analyzed during the current study are available from the corresponding author on reasonable request.

## References

[1] Carrasco TG, Mazzetto MO, Mazzetto RG, Mestriner W (2008). Low intensity laser therapy in temporomandibular disorder: A phase II double-blind study. Cranio.

[2] Herpich CM (2015). Analysis of laser therapy and assessment methods in the rehabilitation of temporomandibular disorder: A systematic review of the literature. J. Phys. Ther. Sci..

[3] Chichorro JG, Porreca F, Sessle B (2017). Mechanisms of craniofacial pain. Cephalalgia.

[4] Vaegter HB, Fehrmann E, Gajsar H, Kreddig N (2020). Endogenous modulation of pain: The role of exercise, stress, and cognitions in humans. Clin. J. Pain..

[5] Monaco A, Cattaneo R, Marci MC, Pietropaoli D, Ortu E (2017). Central sensitization-based classification for temporomandibular disorders: A pathogenetic hypothesis. Pain Res. Manag..

[6] Schiffman E, Ohrbach R (2016). Executive summary of the diagnostic criteria for temporomandibular disorders for clinical and research applications. J. Am. Dent. Assoc..

[7] Abbasgholizadeh ZS, Evren B, Ozkan Y (2020). Evaluation of the efficacy of different treatment modalities for painful temporomandibular disorders. Int. J. Oral Maxillofac. Surg..

[8] Amaral AP (2013). Immediate effect of nonspecific mandibular mobilization on postural control in subjects with temporomandibular disorder: A single-blind, randomized, controlled clinical trial. Braz. J. Phys. Ther..

[9] Machado BC, Mazzetto MO, Da Silva MA, de Felício CM (2016). Effects of oral motor exercises and laser therapy on chronic temporomandibular disorders: A randomized study with follow-up. Lasers Med. Sci..

[10] Seifi M (2017). Comparative effectiveness of low level laser therapy and transcutaneous electric nerve stimulation on temporomandibular joint disorders. J. Lasers Med. Sci..

[11] Venancio RA, Camparis CM, Lizarelli RF (2005). Low intensity laser therapy in the treatment of temporomandibular disorders: A double-blind study. J. Oral Rehabil..

[12] Carvalho CM (2010). Wavelength effect in temporomandibular joint pain: A clinical experience. Lasers Med. Sci..

[13] Marini I, Gatto MR, Bonetti GA (2010). Effects of superpulsed low-level laser therapy on temporomandibular joint pain. Clin. J. Pain..

[14] Prindeze NJ, Moffatt LT, Shupp JW (2012). Mechanisms of action for light therapy: A review of molecular interactions. Exp. Biol. Med..

[15] Ferreira LA, de Oliveira RG, Guimarães JP, Carvalho AC, De Paula MV (2013). Laser acupuncture in patients with temporomandibular dysfunction: A randomized controlled trial. Lasers Med. Sci..

[16] Conti PC (1997). Low level laser therapy in the treatment of temporomandibular disorders (TMD): A double-blind pilot study. Cranio.

[17] Emshoff R, Bösch R, Pümpel E, Schöning H, Strobl H (2008). Low-level laser therapy for treatment of temporomandibular joint pain: A double-blind and placebo-controlled trial. Oral Surg. Oral Med. Oral Pathol. Oral Radiol. Endod..

[18] Geber C (2007). Psychophysics, flare, and neurosecretory function in human pain models: Capsaicin versus electrically evoked pain. J. Pain..

[19] Pigg M, Baad-Hansen L, Svensson P, Drangsholt M, List T (2010). Reliability of intraoral quantitative sensory testing (QST). Pain.

[20] Baad-Hansen L (2015). Reliability of intra-oral quantitative sensory testing (QST) in patients with atypical odontalgia and healthy controls—A multicentre study. J. Oral Rehabil..

[21] Barón J (2017). Differences in topographical pressure pain sensitivity maps of the scalp between patients with migraine and healthy controls. Headache.

[22] Fernández-de-las-Peñas C (2008). Bilateral pressure pain sensitivity mapping of the temporalis muscle in chronic tension-type headache. Headache.

[23] Tang ZT (2018). Reliability of mechanical sensitivity mapping in the orofacial region of healthy Chinese individuals: Towards standardized assessment of somatosensory function. J. Oral Facial Pain Headache..

[24] Axel, B. & Ulrich, L. Syntax of referencing. In *TMJ Disorders and Orofacial Pain: The Role of Dentistry in a Multidisciplinary Diagnostic Approach*, Vol. 32, No 3, 212 (2003).

[25] Brochado FT (2018). Comparative effectiveness of photobiomodulation and manual therapy alone or combined in TMD patients: A randomized clinical trial. Braz. Oral Res..

[26] Rodrigues JH (2015). Evaluation of pain, jaw movements, and psychosocial factors in elderly individuals with temporomandibular disorder under laser phototherapy. Lasers Med. Sci..

[27] Ahrari F, Madani AS, Ghafouri ZS, Tunér J (2014). The efficacy of low-level laser therapy for the treatment of myogenous temporomandibular joint disorder. Lasers Med. Sci..

[28] Da Silva MM (2018). Randomized, blinded, controlled trial on effectiveness of photobiomodulation therapy and exercise training in the fibromyalgia treatment. Lasers Med. Sci..

[29] Mazzetto MO, Hotta TH, Pizzo RC (2010). Measurements of jaw movements and TMJ pain intensity in patients treated with GaAlAs laser. Braz. Dent. J..

[30] Magri LV, Carvalho VA, Rodrigues FCC, Bataglion C, Leite-Panissi CRA (2018). Non-specific effects and clusters of women with painful TMD responders and non-responders to LLLT: Double-blind randomized clinical trial. Lasers Med. Sci..

[31] Shobha R, Narayanan VS, Jagadish Pai BS, Jaishankar HP, Jijin MJ (2017). Low-level laser therapy: A novel therapeutic approach to temporomandibular disorder—A randomized, double-blinded, placebo-controlled trial. Indian J. Dent. Res..

[32] Cavalcanti MF (2016). Comparative study of the physiotherapeutic and drug protocol and low-level laser irradiation in the treatment of pain associated with temporomandibular dysfunction. Photomed. Laser Surg..

[33] Bjordal JM, Johnson MI, Iversen V, Aimbire F, Lopes-Martins RA (2006). Low-level laser therapy in acute pain: A systematic review of possible mechanisms of action and clinical effects in randomized placebo-controlled trials. Photomed. Laser Surg..

[34] Bromm B (1993). The infrared laser in the diagnosis of normal and disturbed pain pathways. Schmerz.

[35] Cotler HB, Chow RT, Hamblin MR, Carroll J (2015). The use of low-level laser therapy (LLLT) for musculoskeletal pain. MOJ Orthop. Rheumatol..

[36] De Freitas LF, Hamblin MR (2016). Proposed mechanisms of photobiomodulation or low-level light therapy. IEEE J. Sel. Top. Quantum Electron..

[37] Hamblin MR (2018). Mechanisms and mitochondrial redox signaling in photobiomodulation. Photochem. Photobiol..

[38] Murad F (2004). Discovery of some of the biological effects of nitric oxide and its role in cell signaling. Biosci. Rep..

[39] Hardie RC (2014). Photosensitive TRPs. Handb. Exp. Pharmacol..

[40] Yang WZ, Chen JY, Yu JT, Zhou LW (2007). Effects of low power laser irradiation on intracellular calcium and histamine release in RBL-2H3 mast cells. Photochem. Photobiol..

[41] Iua V, Klebanov GI, Borisenko GG, Osipov AN (2004). Molecular and cellular mechanisms of the low intensity laser radiation effect. Biofizika.

[42] Chen AC (2011). Low-level laser therapy activates NF-kB via generation of reactive oxygen species in mouse embryonic fibroblasts. PLoS ONE.

[43] Woodruff LD, Bounkeo JM, Brannon WM, Dawes KS, Barham CD (2004). The efficacy of laser therapy in wound repair: A meta-analysis of the literature. Photomed. Laser Surg..

[44] Huang YY, Chen AC, Carroll JD, Hamblin MR (2009). Biphasic dose response in low level light therapy. Dose Response..

[45] Tunér J, Hosseinpour S, Fekrazad R (2019). Photobiomodulation in temporomandibular disorders. Photobiomodul. Photomed. Laser Surg..

[46] Magri LV, Carvalho VA, Rodrigues FC, Bataglion C, Leite-Panissi CR (2017). Effectiveness of low-level laser therapy on pain intensity, pressure pain threshold, and SF-MPQ indexes of women with myofascial pain. Lasers Med. Sci..

[47] Chen J, Huang Z, Ge M, Gao M (2015). Efficacy of low-level laser therapy in the treatment of TMDs: A meta-analysis of 14 randomised controlled trials. J. Oral Rehabil..

[48] Yang G, Baad-Hansen L, Wang K, Xie QF, Svensson P (2014). A study on variability of quantitative sensory testing in healthy participants and painful temporomandibular disorder patients. Somatosens Mot. Res..

[49] Jones DH, Kilgour RD, Comtois AS (2007). Test-retest reliability of pressure pain threshold measurements of the upper limb and torso in young healthy women. J. Pain..

[50] Rolke R, Andrews CK, Magerl W, Treede RD (2005). Deep pain thresholds in the distal limbs of healthy human subjects. Eur. J. Pain..

[51] Thygesen TH, Nørholt SE, Jensen J, Svensson P (2007). Spatial and temporal assessment of orofacial somatosensory sensitivity: A methodological study. J. Orofac. Pain..

[52] Venezian GC, da Silva MA, Mazzetto RG, Mazzetto MO (2010). Low level laser effects on pain to palpation and electromyographic activity in TMD patients: A double-blind, randomized, placebo-controlled study. Cranio.

[53] Da Cunha LA, Firoozmand LM, da Silva AP, Camargo SE, Oliveira W (2008). Efficacy of low-level laser therapy in the treatment of temporomandibular disorder. Int. Dent. J..

[54] da Silva MM, Albertini R, de Carvalho PD (2018). Randoomized, blinded, controlled trial on effectiveness of photobiomodulation therapy and exercise training in the fibromyalgia treatment. Lasers Med. Sci..

[55] Magri LV, Carvalho VA, Rodrigues FCC, Bataglion C, Leite-Panissi CRA (2018). Non-specific effects and clusters of women with painful TMD responders and non-responders to LLLT: Double-blind randomized clinical trial. Lasers Med. Sci..

[56] Shobha R, Narayanan VS, Pai BJ, Jaishankar H, Jijin M (2017). Low-level laser therapy: A novel therapeutic approach to temporomandibular disorder—a randomized, double-blinded, placebo-controlled trial. Indian J. Dent. Res..

[57] Benedetti F, Pollo A, Colloca L (2007). Opioid-mediated placebo responses boost pain endurance and physical performance: Is it doping in sport competitions?. J. Neurosci..

[58] Wager TD (2004). Placebo-induced changes in FMRI in the anticipation and experience of pain. Science.

[59] Eippert F, Finsterbusch J, Bingel U, Büchel C (2009). Direct evidence for spinal cord involvement in placebo analgesia. Science.

[60] Lim PF, Smith S, Bhalang K, Slade GD, Maixner W (2010). Development of temporomandibular disorders is associated with greater bodily pain experience. Clin. J. Pain..

[61] Chouchou F, Lavigne GJ (2014). Placebo analgesia and sleep. Pathol. Biol..

[62] Gourion D, Mouchabac S (2016). Placebo effect: Clinical, biological and therapeutical involvements in depression. Encephale..

[63] Iyomasa MM (2013). Zymographic and ultrastructural evaluations after low-level laser irradiation on masseter muscle of HRS/J strain mice. Lasers Med Sci..

[64] Desiderá AC, Nascimento GC, Gerlach RF, Leite-Panissi CR (2015). Laser therapy reduces gelatinolytic activity in the rat trigeminal ganglion during temporomandibular joint inflammation. Oral Dis..

[65] Schiffman E (2014). Diagnostic criteria for temporomandibular disorders (DC/TMD) for clinical and research applications: Recommendations of the International RDC/TMD consortium network* and orofacial pain special interest group†. J. Oral Facial Pain Headache..

[66] Bisset LM, Evans K, Tuttle N (2015). Reliability of 2 protocols for assessing pressure pain threshold in healthy young adults. J. Manipulative Physiol. Ther..

[67] Svensson P, Graven-Nielsen T, Arendt-Nielsen L (1998). Mechanical hyperesthesia of human facial skin induced by tonic painful stimulation of jaw muscles. Pain.

[68] Ridding MC, Brouwer B, Miles TS, Pitcher JB, Thompson PD (2000). Changes in muscle responses to stimulation of the motor cortex induced by peripheral nerve stimulation in human subjects. Exp. Brain Res..

[69] Shannon CE (1997). The mathematical theory of communication. MD Comput..

[70] Castrillon EE (2017). Entropy of masseter muscle pain sensitivity: A new technique for pain assessment. J. Oral Facial Pain Headache..

