# Supplementary figures and images for: Effect of photobiomodulation therapy on painful temporomandibular disorders
Source: Sci Rep. 2021 Apr 27;11:9049. doi: 10.1038/s41598-021-87265-0 (PMC8079391; doi:10.1038/s41598-021-87265-0)

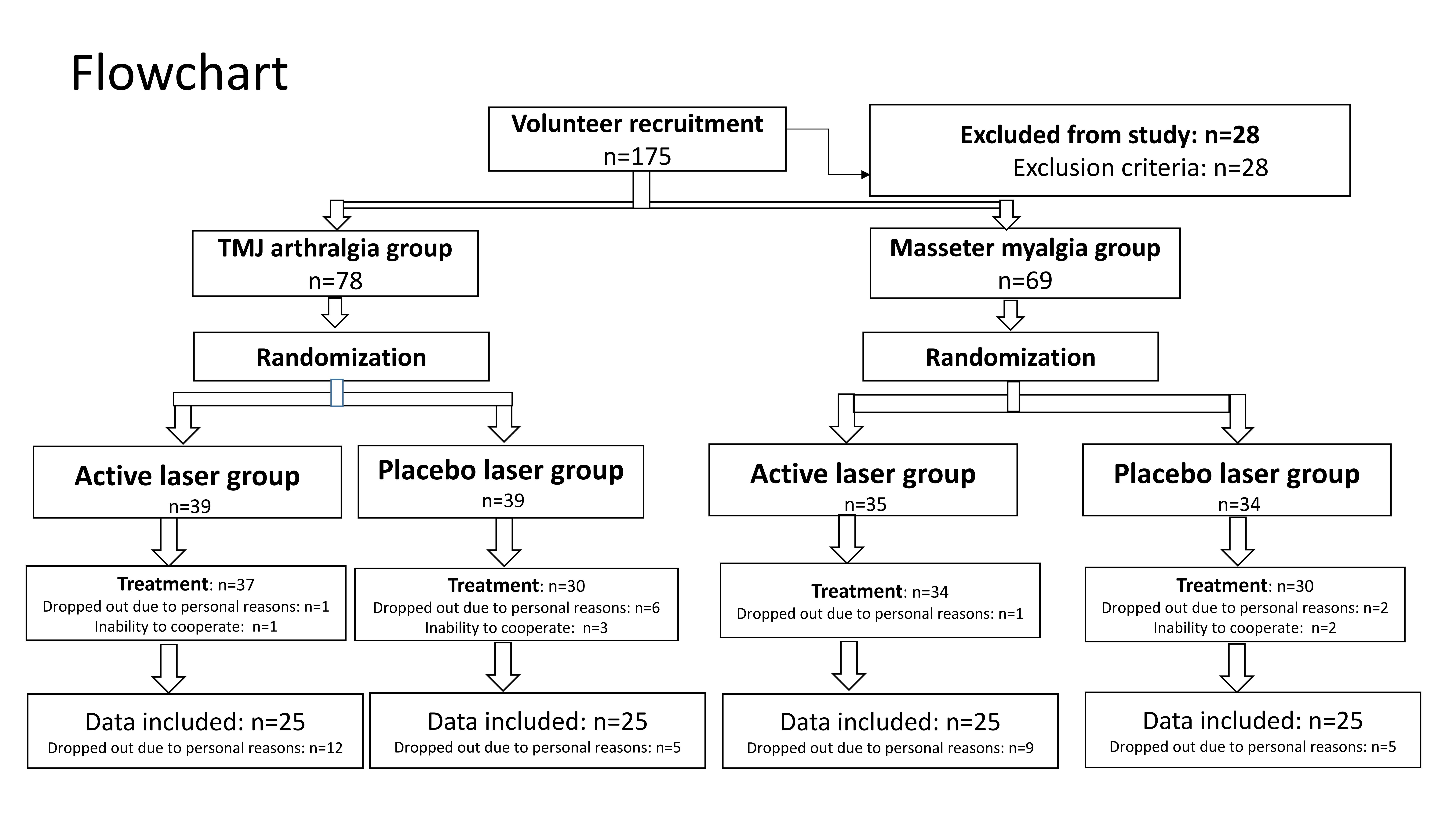

Supplement: Supplementary file 1 — Supplementary Figure S1. [file 41598_2021_87265_MOESM1_ESM.jpg]

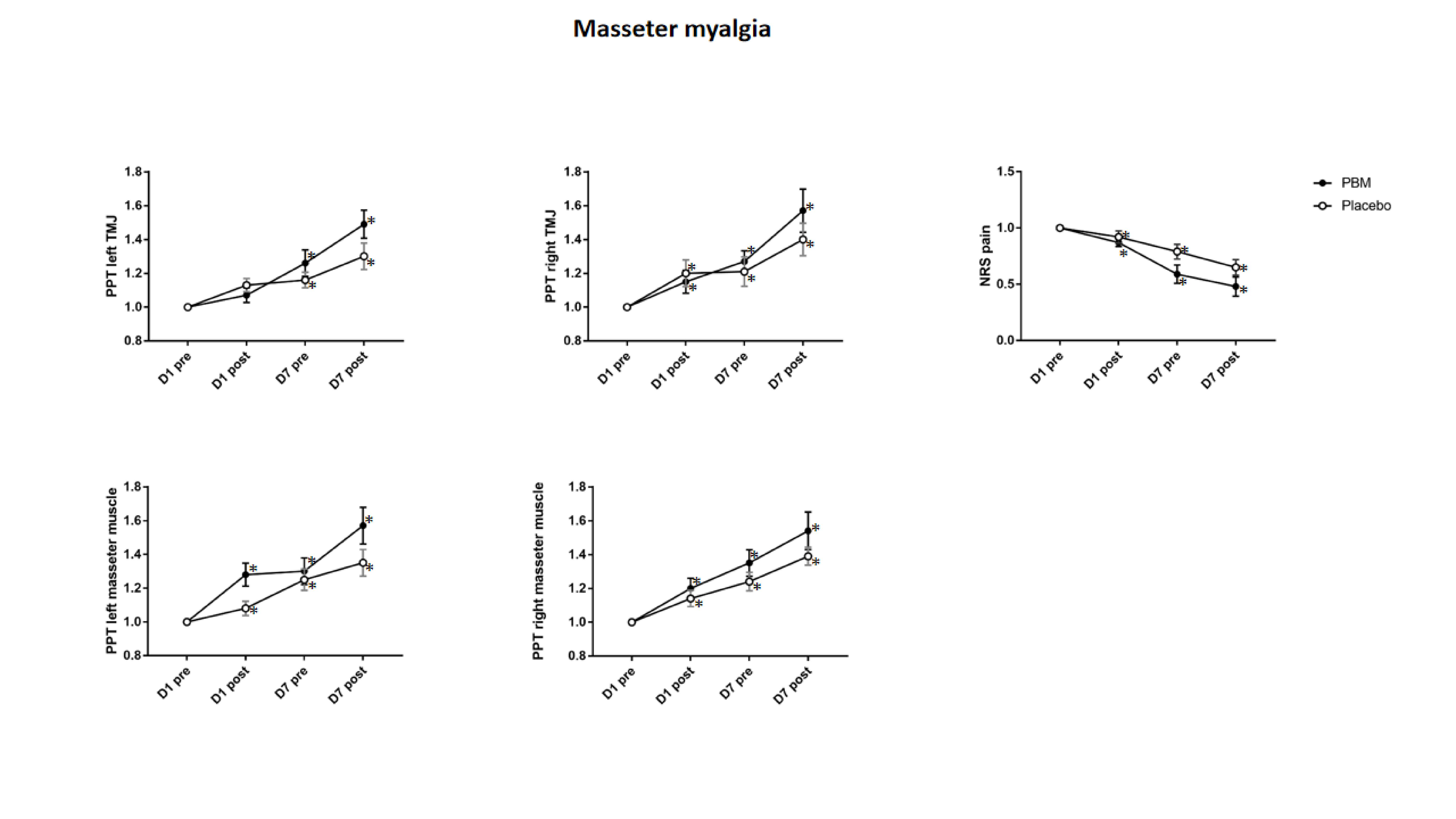

Supplement: Supplementary file 2 — Supplementary Figure S2a. [file 41598_2021_87265_MOESM2_ESM.jpg]

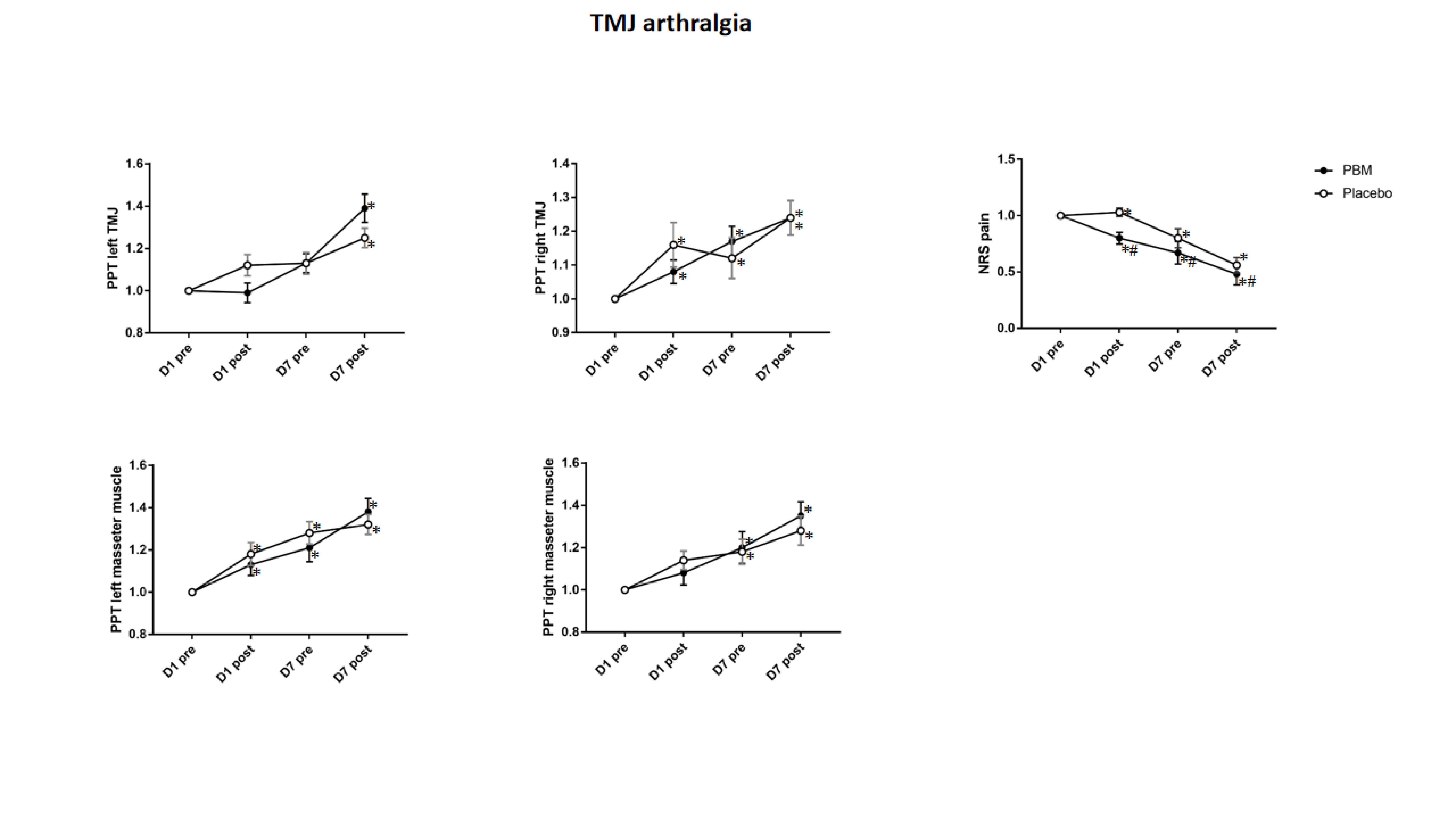

Supplement: Supplementary file 3 — Supplementary Figure S2b. [file 41598_2021_87265_MOESM3_ESM.jpg]

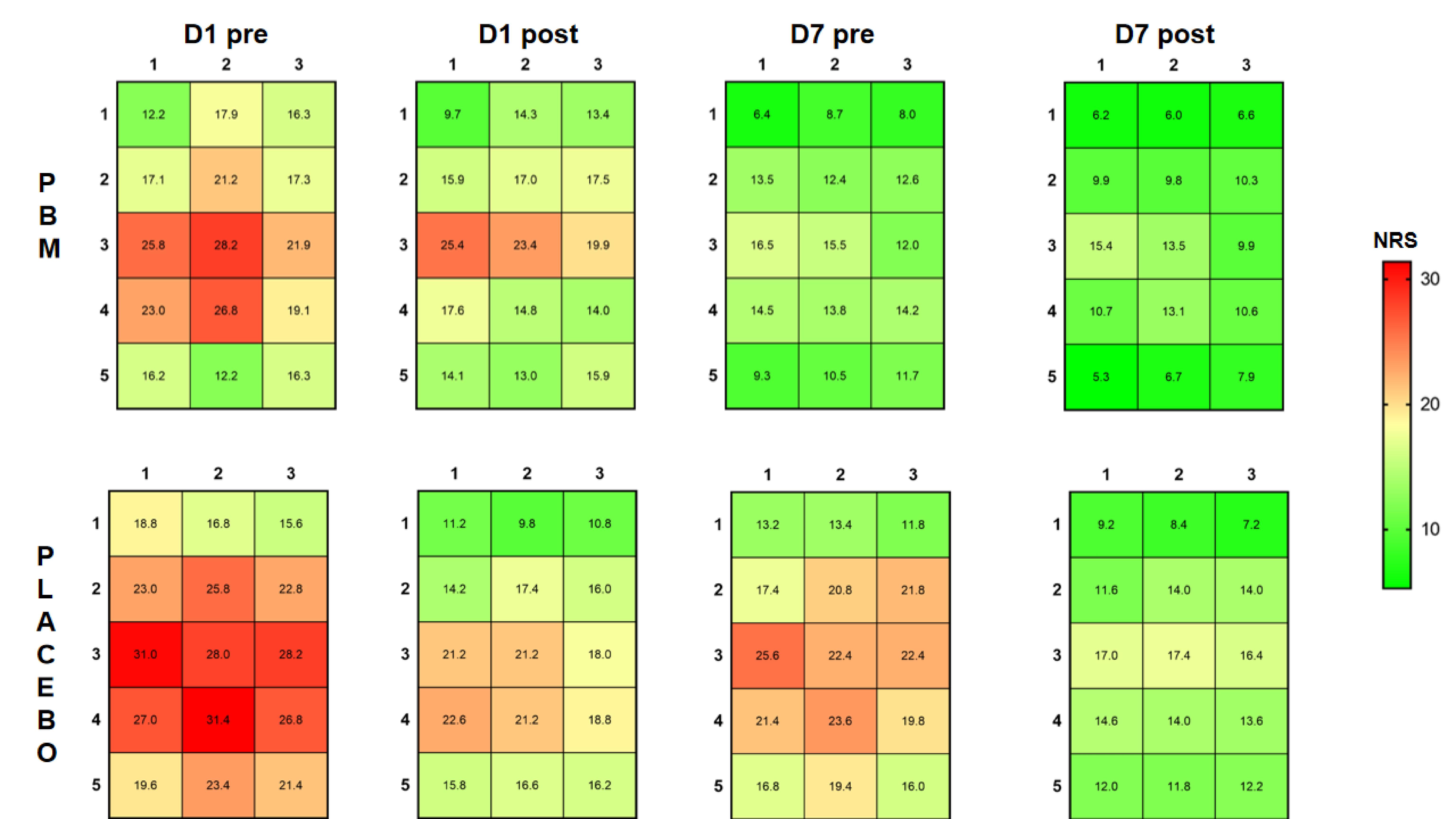

Supplement: Supplementary file 4 — Supplementary Figure S3. [file 41598_2021_87265_MOESM4_ESM.jpg]

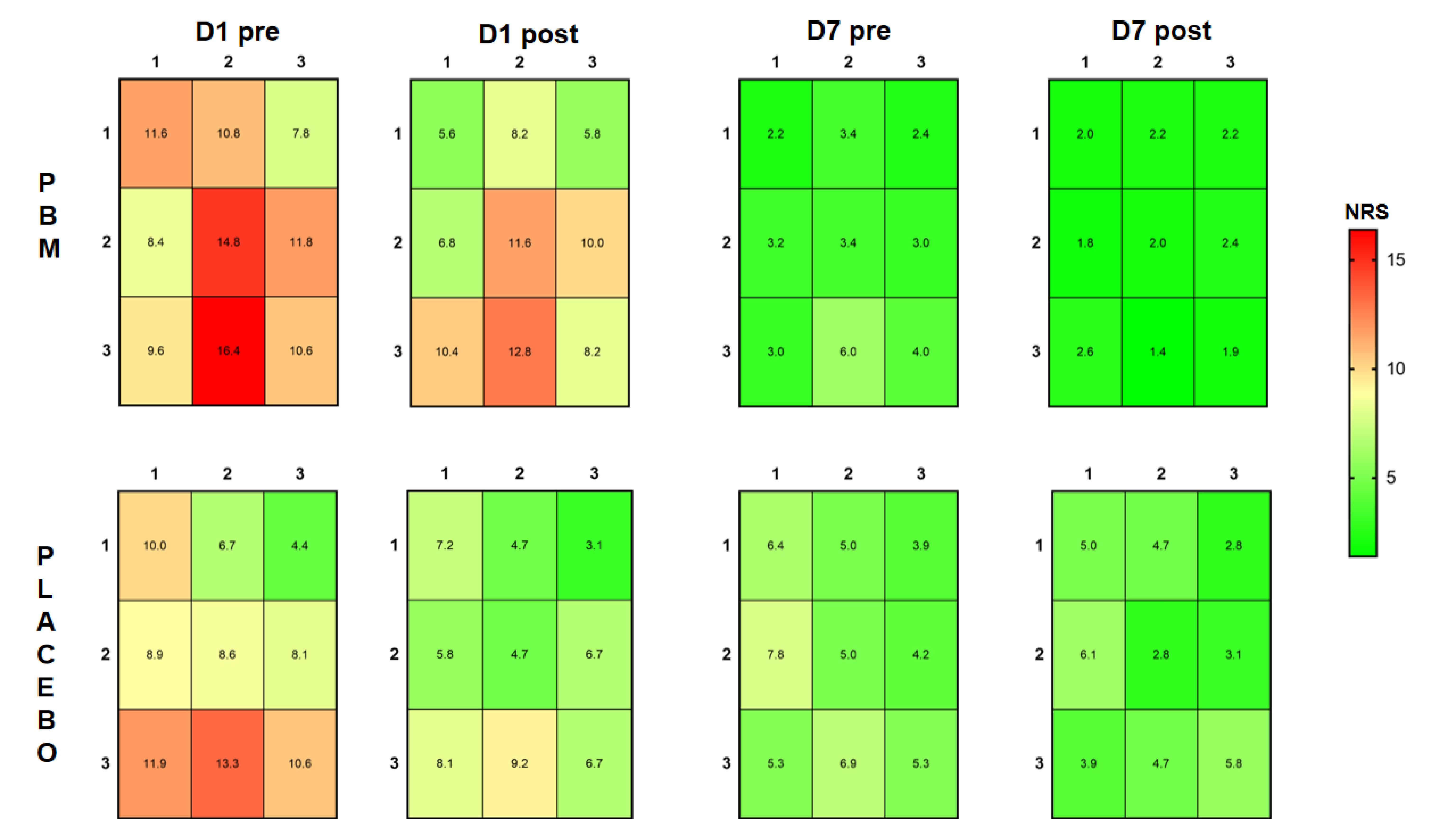

Supplement: Supplementary file 5 — Supplementary Figure S4. [file 41598_2021_87265_MOESM5_ESM.jpg]

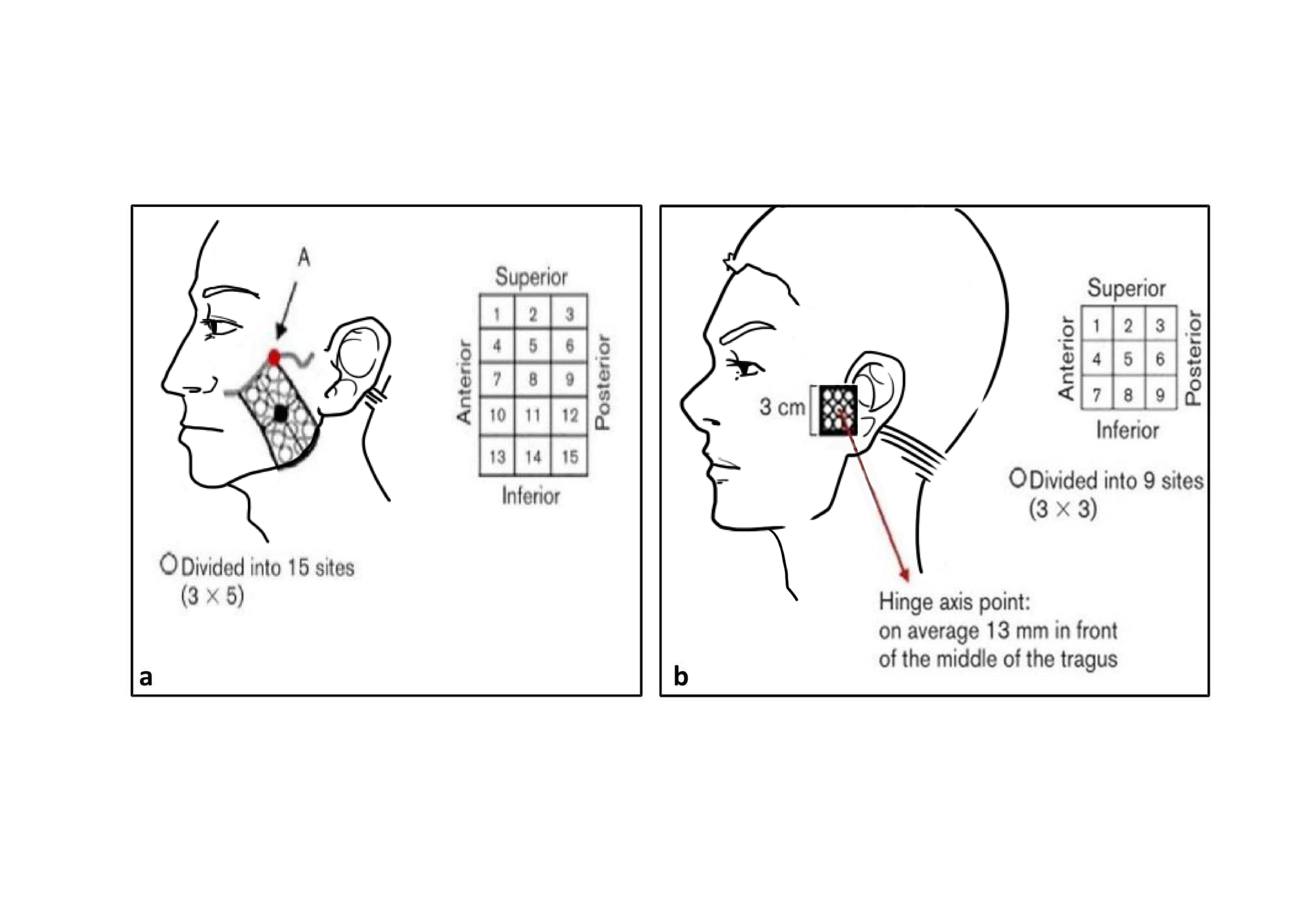

Supplement: Supplementary file 6 — Supplementary Figure S5. [file 41598_2021_87265_MOESM6_ESM.jpg]
